# Changes in Energy Reserves and Gene Expression Elicited by Freezing and Supercooling in the Antarctic Midge, *Belgica antarctica*

**DOI:** 10.3390/insects11010018

**Published:** 2019-12-24

**Authors:** Nicholas M. Teets, Emma G. Dalrymple, Maya H. Hillis, J. D. Gantz, Drew E. Spacht, Richard E. Lee, David L. Denlinger

**Affiliations:** 1 Department of Entomology, University of Kentucky, Lexington, KY 40546, USA; emma.dalrymple@uky.edu (E.G.D.); mhhi222@uky.edu (M.H.H.); 2 Biology Department, Hendrix College, Conway, AK 72032, USA; gantz@hendrix.edu; 3 Department of Evolution, Ecology and Organismal Biology, Ohio State University, Columbus, OH 43210, USA; spacht.2@buckeyemail.osu.edu (D.E.S.); denlinger.1@osu.edu (D.L.D.); 4 Department of Biology, Miami University, Oxford, OH 45056, USA; leere@miamioh.edu; 5 Department of Entomology, Ohio State University, Columbus, OH 43210, USA

**Keywords:** Antarctica, freeze-tolerance, energy stores, heat shock proteins, *Belgica antarctica*

## Abstract

Freeze-tolerance, or the ability to survive internal ice formation, is relatively rare among insects. Larvae of the Antarctic midge *Belgica antarctica* are freeze-tolerant year-round, but in dry environments, the larvae can remain supercooled (i.e., unfrozen) at subzero temperatures. In previous work with summer-acclimatized larvae, we showed that freezing is considerably more stressful than remaining supercooled. Here, these findings are extended by comparing survival, tissue damage, energetic costs, and stress gene expression in larvae that have undergone an artificial winter acclimation regime and are either frozen or supercooled at −5 °C. In contrast to summer larvae, winter larvae survive at −5 °C equally well for up to 14 days, whether frozen or supercooled, and there is no tissue damage at these conditions. In subsequent experiments, we measured energy stores and stress gene expression following cold exposure at −5 °C for either 24 h or 14 days, with and without a 12 h recovery period. We observed slight energetic costs to freezing, as frozen larvae tended to have lower glycogen stores across all groups. In addition, the abundance of two heat shock protein transcripts, *hsp60* and *hsp90*, tended to be higher in frozen larvae, indicating higher levels of protein damage following freezing. Together, these results indicate a slight cost to being frozen relative to remaining supercooled, which may have implications for the selection of hibernacula and responses to climate change.

## 1. Introduction

Cold tolerance is an important determinant of insect species distributions [1]. Insects are often categorized by their ability to survive internal freezing; freeze-tolerant insects can withstand ice formation in the extracellular fluid, while freeze-avoiding species survive subzero conditions by supercooling (i.e., remaining unfrozen below the body fluid melting point) and succumb to internal ice formation [2,3]. More recently, freeze-avoiding species have been further classified into chill-susceptible or chill-tolerant species, depending on their relative sensitivity to low temperature [1,4]. These categories provide a framework for physiological studies of insect cold tolerance, and knowledge of an insect’s cold tolerance strategy is important for predicting responses to future climate scenarios. 

Among cold tolerance strategies, freeze-tolerance is relatively rare, and none of the well-studied insect models (e.g., *Drosophila, Tribolium, Bombyx,* etc.) are capable of surviving freezing. Freeze tolerance appears to have evolved several times in the insect phylogeny [5], and it is more common in the Southern Hemisphere, likely due to increased thermal variability at these latitudes [6]. In addition to the physiological challenges associated with low temperature, freezing provides additional difficulties, including cellular dehydration, mechanical damage from ice formation, and anoxia-reperfusion [5]. While the mechanisms of freeze-tolerance are not well-characterized, recent studies have begun to identify molecular responses that correlate with freezing. In the freeze-tolerant cricket *Gryllus veletis,* cold acclimation that enhances freeze tolerance is associated with the accumulation of several cryoprotectants [7] and upregulation of genes involved in membrane and cytoskeletal remodeling, cryoprotectant transport, molecular chaperone function, and antioxidant responses [8]. In the fly *Chymomyza costata,* which is capable of surviving immersion in liquid nitrogen, cold acclimation is associated with increased expression of genes responsible for protein folding and processing, suggesting that the ability to maintain proteostasis is an important component of freeze tolerance [9]. 

In Antarctica’s terrestrial climates, the threat of freezing is year-round, and the arthropods that live there are well-adapted to the cold. Mites and Collembola, which are the most speciose of the terrestrial arthropods in Antarctica, are freeze-avoidant but have an impressive ability to supercool (with supercooling points at or below −30 °C for most species) [10]. The three insect species in Antarctica, all chironomids, likely overwinter as larvae. The midge *Parachlus steinenii* is aquatic and has limited cold tolerance, with a lower lethal temperature around −3 °C for summer larvae, which is well above the supercooling point [11]. In contrast, the other two midges, *Belgica antarctica* and the closely related invasive species *Eretmoptera murphyi,* are freeze tolerant and can survive in the frozen state down to around −20 °C [12,13,14,15].

The midge *B. antarctica* is the world’s southernmost insect and is endemic to Antarctica, making it an excellent system for studying the mechanistic basis of adaptation to polar environments. While larvae of this species are freeze-tolerant, the likelihood of freezing depends on environmental conditions. Depending on the time of year, the supercooling point of larvae is typically between −5 and −15 °C [16,17,18], which is below the typical microhabitat temperature, even in winter [18,19]. While the risk of spontaneous freezing is low, larvae have permeable cuticles and are highly susceptible to inoculative freezing (i.e., internal freezing caused by direct contact with environmental ice), so they likely freeze at high subzero temperatures in many of their habitats [20]. However, in drier conditions, larvae do have a limited ability to withstand inoculative freezing and can either remain supercooled or undergo cryoprotective dehydration [19]. In these relatively dry environments, cryoprotective dehydration likely occurs when chilling is gradual, but if the rate of cooling exceeds the ability of larvae to dehydrate, larvae can remain supercooled, at least for short periods of time. Thus, it appears *B. antarctica* has a flexible cold tolerance strategy and survives sub-zero temperatures by freezing, supercooling, or cryoprotective dehydration, depending on hygric conditions and the duration of cold exposure. 

Previous studies on *B. antarctica* suggest that freezing is more stressful than remaining supercooled in summer-acclimatized larvae. After being subjected to repeated cold exposure at −5 °C, frozen larvae experience higher mortality, higher levels of tissue damage, reduced energy stores, and higher mRNA expression of a stress protein (hsp70) relative to the supercooled larvae [21]. A similar comparison of long-term freezing and cryoprotective dehydration saw no differences in survival between the two phenotypes, but frozen larvae experience more pronounced depletion of glycogen stores [18]. Thus, while inoculative freezing is unavoidable in some environments, freezing increases the risk of mortality and is more energetically costly than remaining unfrozen. Our previous work also showed that frozen larvae have higher expression of a 70 kDa heat shock protein, suggesting a higher degree of protein damage. In the time since, we have sequenced the genome of *B. antarctica* [22], allowing for thorough tests of the hypothesis that freezing and supercooling elicit distinct molecular responses. 

Here, the physiological costs of freezing are assessed by comparing responses to freezing and supercooling in larvae prepared for winter conditions. Our objectives were to (1) assess survival and tissue damage in winter-acclimated larvae that are frozen and supercooled at the same temperature, (2) quantify the energetic costs of freezing and supercooling by measuring levels of key energy stores, and (3) test the hypothesis that freezing and supercooling elicit distinct cell stress responses by measuring transcript levels of heat shock proteins from each of the five major families. Heat shock proteins are a class of molecular chaperones that are upregulated in direct response to protein denaturation [23], and expression of these genes has been used as a biomarker for sublethal stress in *B. antarctica* [24]. Our experiments indicate that while winter-acclimated larvae survive freezing and supercooling equally well, freezing results in slightly greater energetic costs and elevated expression of certain heat shock proteins.

## 2. Materials and Methods 

### 2.1. Insects

Larvae of *B. antarctica* were collected from various offshore islands in the vicinity of Palmer Station (64°46′ S, 64°04′ W) in February and March 2017. Substrate samples containing larvae were returned to the station, and larvae were extracted into ice water using a modified Berlese apparatus. After extraction, larvae were returned to pans containing natural substrate (rocks, moss, and the alga *Prasiola crispa*) and stored at 2 °C. Samples were shipped back to the United States in a container maintained between 0 and −5 °C for ~3 weeks, after which they were stored in our home laboratory at 4 °C. Experiments for this study were conducted between July and October 2017. The natural phenology of *B. antarctica,* coupled with the process of shipping at low temperature, induced winter acclimation in larvae, such that larvae tested in July were considerably more cold-tolerant than freshly collected larvae tested in January to February, e.g., reference [16]. Thus, for simplicity, these larvae are referred to as winter-acclimated, in contrast with summer-acclimatized larvae used in our previous comparison of freezing and supercooling [21]. 

Prior to experimentation, larvae were sorted from their substrate in ice water and kept on a moist paper towel overnight to facilitate gut clearance. Fourth instar larvae were used for all experiments. 

### 2.2. Temperature Treatments

For these experiments, all cold treatments occurred at −5 °C in a programmable refrigerated bath (Arctic A24B, ThermoFisher Scientific, Waltham, MA, USA). Larvae were exposed to cold in groups of 10–20 in 1.5 mL microcentrifuge tubes. For the frozen group, 50 µL of ddH_2_O and a small piece of ice were added to each tube to ensure that larvae froze via inoculative freezing. For the supercooled group, larvae were blotted dry with a Kimwipe and added to a dry microcentrifuge tube. To ensure larvae can remain supercooled for the duration of the experiment, 8 larvae were blotted dry, affixed to a type T thermocouple, and held at −5 °C for 14 days. No freezing exotherms were observed, indicating that the supercooled state was stable. 

### 2.3. Larval Survival after Cold Exposure

To test the hypothesis that freezing leads to higher mortality than remaining supercooled, groups of 20 larvae were either frozen or supercooled at −5 °C for up to 14 days. Samples were removed every 2 days to check survival. After cold treatment, excess water was removed from the frozen tubes, and a drop of water was added to the supercooled tubes to prevent desiccation. Larvae were then given 24 h recovery at 4 °C before assessing survival. To assess survival, larvae were placed in a Petri dish with ice water and observed under a microscope. Larvae that moved spontaneously or in response to gentle prodding were considered alive. For each group, N = 3 replicates of 20 larvae. In this experiment, there was no significant mortality observed from the cold (see Results), thus for subsequent experiments on sublethal measures of stress, we opted to expose larvae to −5 °C for either 24 h or 14 days, to assess the consequences of short- and long-term freezing. 

### 2.4. Cell Survival after Cold Exposure

Tissue damage was quantified after cold exposure using a two-component dye exclusion assay that distinguished between the live and dead cells. This assay was modified from the LIVE/DEAD Sperm Viability Kit (ThermoFisher Scientific, Waltham, MA), according to Yi and Lee (2003) [25]. Larvae were frozen or supercooled at −5 °C for 24 h or 14 days, after which they recovered for 24 h at 4 °C. Control samples were maintained at 4 °C and measured with the 24 h groups. After recovery, midgut and fat body tissue were dissected in the ice-cold Coast’s solution [26] and transferred to a slide containing 25 µL SYBR-14 dye in Coast’s solution. After 10 min, 25 µL propidium iodide in Coast’s solution was added to the slide, and samples were incubated at room temperature for an additional 10 min. A coverslip was placed on each sample, and tissues were imaged on a fluorescence microscope using the FITC and Texas Red filters. Green (SYBR-14) and red (propidium iodide) images were overlaid, and survival was assessed by counting the proportion of live (green) and dead (red) nuclei in a representative area containing 300 cells. For each treatment and tissue, N = 5 independent replicates. 

### 2.5. Energy Store Analyses 

Carbohydrate and lipid energy stores were measured using colorimetric assays, as described previously [21]. Groups of 10 larvae were frozen or supercooled at −5 °C for 24 h or 14 days and sampled either immediately after treatment or after 12 h recovery at 4 °C. In previous work, some biochemical and molecular responses to the cold occurred specifically during recovery [21,27], which was why these recovery groups were included. Control larvae were maintained at 4 °C and sampled at the onset of the experiment. After treatment, groups of larvae were weighed and stored at −80 °C until the time of analysis. Carbohydrates were extracted from groups of 10 larvae using perchloric acid, and the samples were neutralized with KOH. Glucose was measured using the Glucose Assay Kit (Sigma-Aldrich, St. Louis, MO, USA) according to the manufacturer’s instructions but scaled down for microplates. While glucose was not a primary energy store for *B. antarctica,* in previous work, mobilization of glucose in response to stress was observed, with the degree of mobilization proportionate to the stressfulness of the treatment [21,24,28]. Glycogen was measured by first treating samples with amyloglucosidase from *Aspergillus niger* (Sigma-Aldrich) at 55 °C for 2 h and then measuring glucose concentration. For trehalose measurements, samples were digested overnight at 37 °C with trehalase from porcine kidney (Sigma-Aldrich) prior to measuring glucose. For lipid analysis, samples were homogenized in 1:1 chloroform methanol, the solvent was evaporated, and lipids were quantified using vanillin-phosphoric acid reagent. For all assays, absorbance was measured in a Clariostar multimode plate reader (BMG Labtech, Cary, NC, USA), and absorbance values were compared to a 7-point standard curve for each metabolite.

### 2.6. Gene Expression Measurements

Following freezing and supercooling, the abundance of 5 heat shock protein transcripts was measured with qPCR on cDNA derived from mRNA. The genome of *B. antarctica* [22] was mined for heat shock protein sequences using BLAST, and we selected one gene from each of the major families of heat shock proteins: Small heat shock proteins (*sHsp*), *hsp40*, *hsp60*, *hsp70*, and *hsp90.* For families with multiple genes, we selected the gene with the highest induction rate from a previous study of dehydration-induced changes in gene expression [29]. Primers were designed to have an annealing temperature of 60 °C and a product size of 100–150 bp using software from Integrated DNA Technologies (Coralville, IA, USA); see Table 1. Primers were validated using an 8-point standard curve to ensure linearity and optimal efficiency. Primer sequences for *rpl19*, the reference gene, were obtained from a previous study [27].

Larvae were exposed to the same treatments described above in Section 2.5, and samples were frozen at −80 °C until RNA extraction. RNA was extracted from groups of 10 larvae using Tri Reagent (ThermoFisher) and further purified with the RiboPure RNA Purification Kit (ThermoFisher). The quantity and purity of RNA were assessed on a spectrophotometer, and cDNA was synthesized from 1 µg RNA using the qScript cDNA Synthesis Kit (Quanta Bio, Beverly, MA, USA). To measure transcript abundance, each 20 µL qPCR reaction contained 10 µL 2x PerfeCTa SYBR Green Fastmix (Quanta Bio), 2 µL each primer at 2.5 µmol L^−1^, 2 µL cDNA, and 4 µL water. Reactions were run for 40 cycles on a QuantStudio 6 Flex real-time PCR system (ThermoFisher), and gene expression was calculated using the 2^−ΔCt^ method as described previously [27]. Transcript abundance was expressed as a log_2_ fold change, which was obtained from the fitted values from linear models (see Statistical Analyses below).

### 2.7. Statistical Analyses 

All statistical analyses were conducted using R statistical software (v. 3.6.0) and JMP Pro 14 (SAS Institute Inc., Cary, NC, USA). Larval and cell survival data were analyzed using a generalized linear model with a binomial error distribution with the glmer function in the lme4 package in R. Larval survival was fit as a function of treatment (frozen or supercooled) and exposure time, with replicate nested within the group as a random effect. Cell survival data were fit as a function of treatment group, with replicate nested within the group as a random effect. Energy store data were analyzed with two different least squares regression models. First, to conduct pairwise comparisons across all 9 experimental groups, a model was fit with the group as a fixed effect and analytical block (samples were run across two microplates) as a random effect. Second, to test for the general effects of treatment (frozen or supercooled), exposure time (24 h or 14 days), and recovery time (0 or 24 h), we omitted the control group and fit a full factorial least squares model with treatment, exposure time, recovery time, and their interactions as fixed effects and analytical block as a random effect. Gene expression data were analyzed in the same manner, fitting separate models for pairwise comparisons and effect tests. For energy stores data, metabolite concentrations were corrected for the weight of the sample prior to analysis. For gene expression data, we used the ΔCt as the response variable. We used the p.adjust function in R to apply a false discovery rate correction to each pairwise comparison and control the type I error rate [30].

## 3. Results

### 3.1. Larval Survival

**Figure 1:**
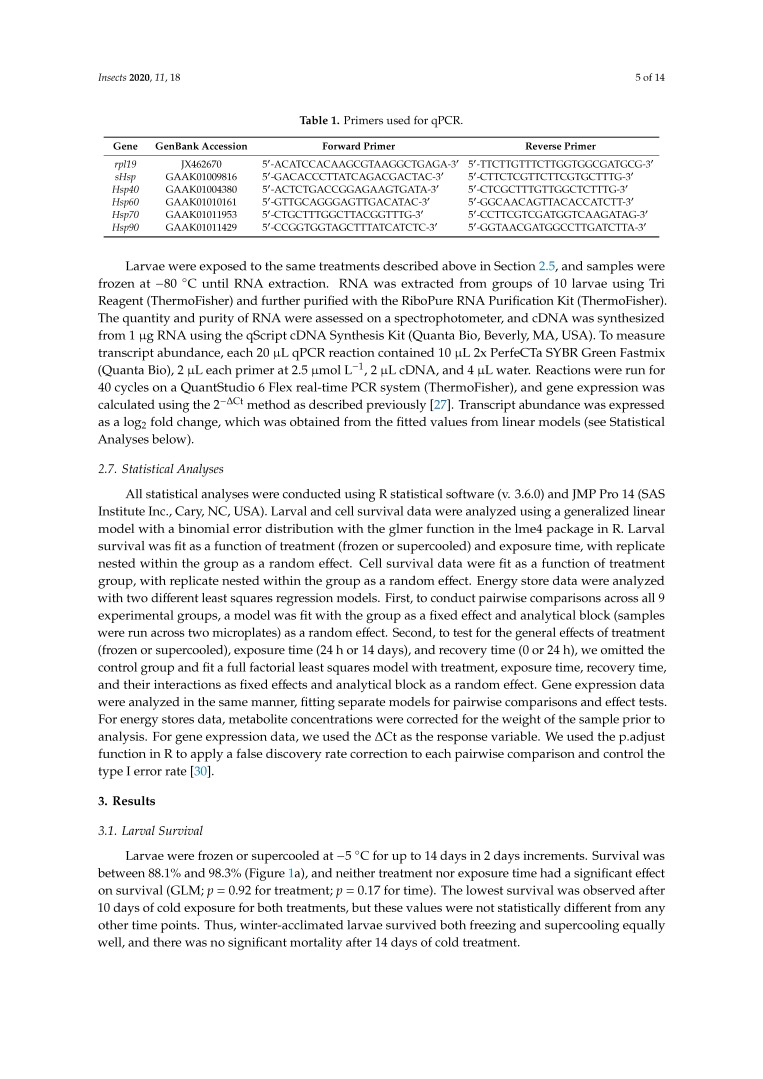
Winter-acclimated larvae survive freezing and supercooling at −5 °C equally well. (**a**) Survival of frozen and supercooled larvae following cold exposure at −5 °C for the indicated time periods. (**b**) Midgut and (**c**) fat body cell survival following freezing and supercooling at −5 °C for 24 h or 14 days. In all panels, symbols represent mean ± s.e.m., N = 3 for a, N = 5 for b and c. In b and c, different letters indicate statistically significant differences between groups (GLM, planned contrast with Tukey correction, *p* < 0.05).

Larvae were frozen or supercooled at −5 °C for up to 14 days in 2 days increments. Survival was between 88.1% and 98.3% (Figure 1a), and neither treatment nor exposure time had a significant effect on survival (GLM; *p* = 0.92 for treatment; *p* = 0.17 for time). The lowest survival was observed after 10 days of cold exposure for both treatments, but these values were not statistically different from any other time points. Thus, winter-acclimated larvae survived both freezing and supercooling equally well, and there was no significant mortality after 14 days of cold treatment. 

### 3.2. Cell Survival

As with larval survival, very little tissue damage was observed in response to freezing and supercooling at −5 °C. Cell survival was measured in midgut and fat body tissues after 24 h and 14 days of cold exposure. In the midgut, cell survival of control samples was 87.1% ± 6.0%, and survival decreased to 76.2% ± 6.5% after 24 h freezing, but this difference was not statistically significant (Figure 1b, GLM, planned contrast with Tukey correction, *p* = 0.34). Cell survival was not different between supercooled and frozen samples at either time point, and survival was indistinguishable from controls. After 14 days of cold exposure, cell survival was nearly identical between frozen and supercooled larvae: 88.2% ± 2.2% for frozen, and 90.0% ± 2.6% for supercooled (Figure 1b). Similar patterns were observed in the fat body, as cell survival was statistically indistinguishable between frozen and supercooled larvae at both time points, and none of the groups were different from controls (Figure 1c). 

### 3.3. Energy Stores

**Figure 2:**
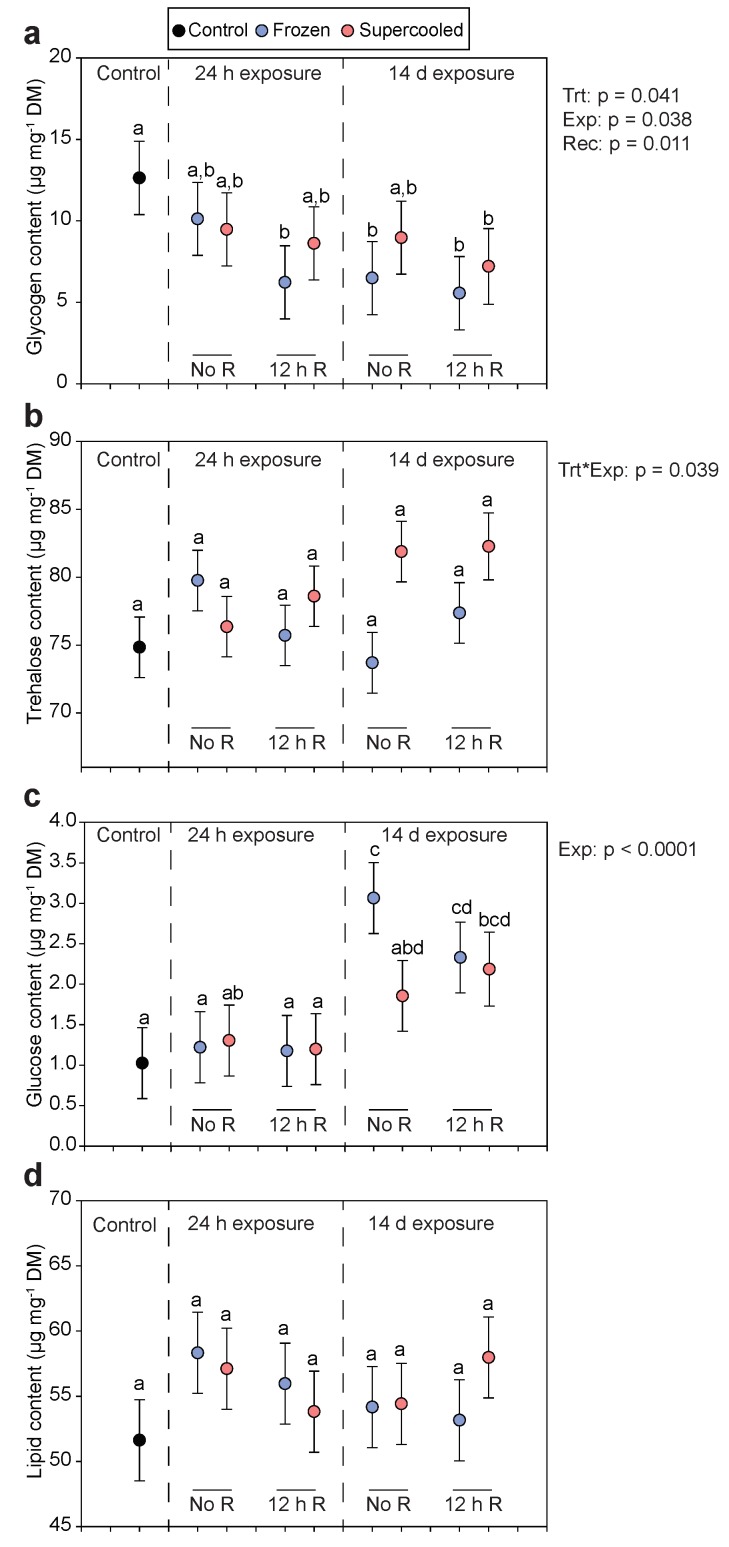
Levels of energy reserves after freezing and supercooling at −5 °C. (**a**) Glycogen, (**b**) trehalose, (**c**) glucose, and (**d**) lipid content of larvae following freezing (blue) or supercooling (red) for either 24 h or 14 days. Larvae were sampled either immediately after treatment (No R) or after 12 h recovery at 4 °C (12 h R). Symbols represent fitted values ± s.e.m. from a linear model fitting metabolite content as a function of the group, with the analytical block as a random effect. For each group, N = 5. Different letters indicate significant a difference based on pairwise comparisons of all groups (Linear Model, planned contrasts with FDR correction, *p* < 0.05). The *p*-values to the right of each panel indicate the significant effects from a full factorial model fitting metabolite content as a function of treatment (frozen or supercooled), exposure time (24 h or 14 days), recovery time (0 or 12 h), and their interactions as fixed effects with analytical block as a random effect. For this model, the control group was omitted so that we could have a full factorial model to isolate effects across groups. In the *y*-axis label, DM = dry mass.

To assess the energetic costs of freezing and supercooling, the levels of carbohydrate and lipid energy stores were measured after cold exposure. Energy stores were measured after 24 h and 14 days of exposure, both immediately after exposure and after 12 h recovery at 4 °C. A major carbohydrate energy store, glycogen, significantly decreased relative to controls after recovery from a 24 h freezing event (Figure 2a; Linear Model, planned contrast with FDR correction, *p* = 0.011), although glycogen levels were statistically indistinguishable between frozen and supercooled larvae. After 14 days cold exposure, glycogen levels in both frozen and supercooled larvae were significantly lower than controls after recovery (Figure 2b). To further analyze the effects of our treatments, exposure time, and recovery time on glycogen levels, we omitted the control group and fitted a full factorial model with treatment (frozen vs. supercooled), exposure time (24 h or 14 days), recovery time (0 or 12 h), and their interactions as fixed effects. In this model, the effects of treatment (*p* = 0.041), exposure time (*p* = 0.038), and recovery time (*p* = 0.012) were all significant. Specifically, glycogen levels tended to be lower in frozen larvae across all groups, and glycogen levels decreased with increased exposure and recovery time (Figure 2a). 

The other primary carbohydrate energy store, trehalose, was largely unaffected by our treatments. Trehalose levels were statistically indistinguishable between any of the groups (Figure 2b; Linear Model, planned contrasts with FDR correction, *p* >0.05), and in the full factorial model, there was a significant interaction between treatment and exposure time (*p* = 0.039), with supercooled larvae receiving 14 days of cold exposure having slightly higher trehalose levels than other groups (Figure 2b). Glucose levels after 24 h cold exposure, with or without recovery, were indistinguishable from controls (Figure 2c). In response to prolonged freezing and supercooling, glucose levels increased and were highest among larvae that were frozen for 14 days with no recovery (Figure 2c). In the full factorial model, the effect of exposure time on glucose levels was highly significant (*p* < 0.0001), but no other effects were statistically significant. Lipid levels were unchanged by any of the treatments (Figure 2d), and none of the effects in the full factorial model were statistically significant.

### 3.4. Gene Expression

**Figure 3:**
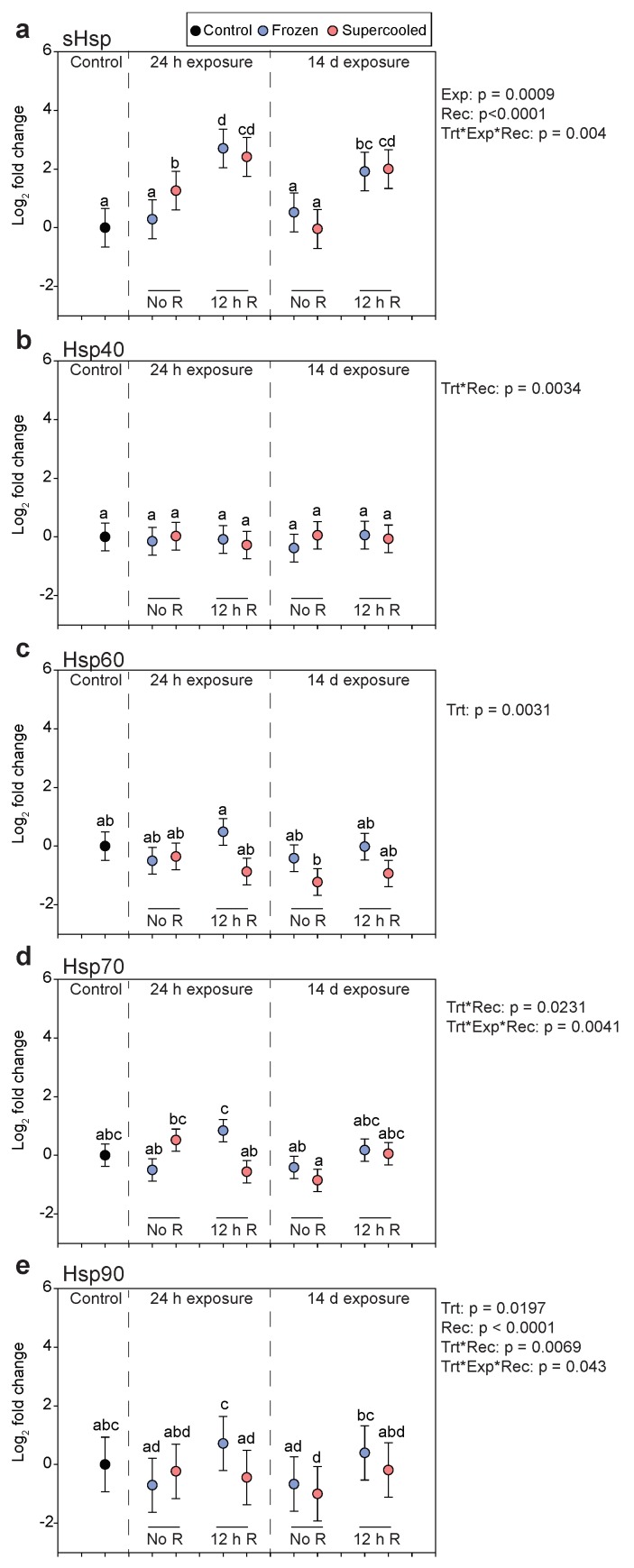
Transcript abundance of heat shock proteins after freezing and supercooling at −5 °C. (**a**) *sHsp*, (**b**) *hsp40*, (**c**) *hsp60*, (**d**) *hsp70,* and (**e**) *hsp90* transcript levels following freezing (blue) or supercooling (red) for either 24 h or 14 days. Larvae were sampled either immediately after treatment (No R) or after 12 h recovery at 4 °C (12 h R). Symbols represent fitted values ± s.e.m. from a linear model fitting ΔCt as a function of the group, with analytical block as a random effect. For each group, N = 5. Different letters indicate significant differences based on pairwise comparisons of all groups (Linear Model, planned contrasts with FDR correction, *p* < 0.05). The *p*-values to the right of each panel indicate the significant effects from a full factorial model fitting ΔCt as a function of treatment (frozen or supercooled), exposure time (24 h or 14 days), recovery time (0 or 12 h), and their interactions as fixed effects with analytical block as a random effect. For this model, the control group was omitted, thus that we could have a full factorial model to isolate effects across groups.

Transcript abundance of *sHsp* increased in response to both freezing and supercooling, primarily during recovery. Relative to controls, transcript levels were slightly elevated after 24 h supercooling (Figure 3a; Linear Model, planned contrast with FDR correction, *p* = 0.00076), and levels were more than four-fold higher than controls in both frozen and supercooled larvae after 12 h recovery (Figure 3a). Transcript abundance increased to nearly the same level following recovery from 24 h cold exposure in both frozen and supercooled larvae. In the full factorial model to test effects across groups, exposure time (*p* = 0.0009) and recovery time (*p* < 0.0001) had significant effects on transcript levels, but transcript levels were not different between frozen and supercooled larvae across groups (*p* = 0.74). Transcript abundance of *hsp40* did not change across groups (Figure 3b), but there was a significant treatment by recovery time interaction in the full factorial model (*p* = 0.0034). For *hsp60,* there were no strong changes in transcript levels across groups, but there was a significant treatment effect (*p* = 0.0031), with frozen larvae tending to have higher expression than supercooled larvae (Figure 3c). 

Transcript levels for *hsp70* were not statistically different from controls at any time point (Figure 3d), but frozen larvae had significantly higher expression than supercooled larvae after recovery from a 24 h cold exposure (linear model, planned contrasts with FDR correction, *p* = 0.031). In addition, the two-way interaction of treatment and recovery time was significant (*p* = 0.0231), as frozen larvae with 12 h recovery tended to have the highest expression, and there was also a significant three-way interaction between treatment, exposure time, and recovery time (*p* = 0.0041). Finally, for *hsp90*, again none of the groups were statistically different from controls in pairwise comparisons (Figure 3e), but as with *hsp70* transcript levels were higher in frozen larvae relative to supercooled larvae after recovery from a 24 h cold exposure (Linear Model, planned contrasts with FDR correction, *p* = 0.0078). The effects of treatment (*p* = 0.0197), recovery time (*p* <0.0001), the treatment by recovery time interaction (*p* = 0.0069), and the three-way interaction between treatment, exposure time, and recovery time (*p* = 0.043) were all significant. In particular, frozen larvae tended to have higher expression across groups, and larvae tended to have higher expression after recovery. 

## 4. Discussion

Larvae of *B. antarctica* have a flexible cold tolerance strategy, in that they can either survive inoculative freezing or remain unfrozen at subzero temperatures, depending on the hygric conditions of their environment [18,19,20,21]. Here, we assessed the survival, tissue damage, energy stores, and stress gene expression in winter-acclimated larvae that were either frozen or supercooled at −5 °C for up to 14 days. To our surprise, larvae survived both conditions equally well, with no significant mortality at either condition (Figure 1a). Further, we did not observe any evidence of sublethal tissue damage, as both midgut and fat body tissue had high cell survival at both conditions (Figure 1b,c). These results are in stark contrast to our previous work on summer acclimatized larvae, in which freezing was clearly more stressful than supercooling [21]. In a previous study, larvae frozen for only 60 h had ~50% survival, compared to ~90% survival after 14 days of freezing in this study, and repeated freezing events at −5 °C caused significant tissue damage in the midgut that was not observed in supercooled larvae. Thus, winter acclimation substantially improves freezing tolerance in these midges.

Notably, larvae were kept at a summer-like temperature of 4 °C in our home laboratory, but the experiments were conducted during the Antarctic winter (July October). Thus, either larvae are ontogenetically programmed to be more freeze-tolerant during the winter, or the low temperatures experienced during shipping (0 to −5 °C for ~3 weeks) lead to a permanent enhancement of freeze-tolerance. In the field, seasonal metabolic depression is intrinsically programmed and occurs in the absence of clear environmental cues [31]. Perhaps enhanced freeze-tolerance accompanies metabolic depression and is also a component of seasonal preparation for winter, but additional experiments with field-collected larvae are needed to test this hypothesis. Regardless, our results clearly show that winter-acclimated larvae survive prolonged periods of cold equally well in both the frozen and supercooled state. 

While larvae and their tissues survived equally well frozen and supercooled, we did observe some evidence of sublethal costs to freezing relative to remaining supercooled. Larvae frozen for 14 days had 56% lower glycogen levels than controls, and across all treatments, frozen larvae tended to have lower glycogen levels than supercooled larvae (Figure 2a). The other two primary energy stores, trehalose, and lipids, were unaffected by freezing (Figure 2b,d), but the pronounced decrease in glycogen levels indicate energetic costs to freezing. Similar sublethal costs to freezing have been observed in other systems. In the goldenrod gall fly, *Eurosta solidaginis,* repeated bouts of freezing and thawing (which are more stressful than a continuous bout of freezing) have no impact on overwintering survival but reduce egg production by ~10% [32]. Similarly, in the sub-Antarctic caterpillar *Pringleophaga marioni,* repeated freeze-thaw is nonlethal but impairs feeding and results in smaller body size [33]. In our previous study with summer-acclimatized larvae, repeated freezing and a single 60 h freezing event also caused glycogen depletion relative to supercooled larvae [21], but the differences between frozen and supercooled larvae were much more pronounced than we observed here. Whether these energetic costs of freezing ultimately impact fitness is uncertain since we are unable to reliably rear larvae to adulthood. However, given the short growing seasons in Antarctica, we suspect that the energy deficits caused by larval freezing may have impacts later in life.

The slight glycogen reduction in frozen larvae may be explained by mobilization of glucose, as frozen larvae had the highest glucose levels after 14 days of cold exposure (Figure 2c). Glucose mobilization during stress is frequently observed in *B. antarctica,* and in previous studies, treatments that lead to higher mortality or higher sublethal injury yield higher rates of glucose mobilization [21,24,28]. During stress, glucose can function as a cryoprotectant or serve as a substrate for the synthesis of cryoprotective sugar alcohols [34], but we did not assess cryoprotectant loads in this study. Mobilization of glucose may also fuel energetic demands of repairing freezing injury, as many of the physiological challenges of freezing (e.g., cellular dehydration, osmotic imbalance, protein damage, oxidative stress, etc.) are energetically costly [5]. In the 24 h freezing group, there was a trend towards continued glycogen depletion during recovery, suggesting investment of energy into repair processes. However, the depletion of glycogen observed in larvae frozen for 2 weeks likely reflects mobilization of glucose for cryoprotectants and other metabolic processes, as we do not expect physiological repair processes to be active while larvae are still frozen. 

The gene expression experiments also indicated slight sublethal costs to freezing. We measured transcript levels of five different heat shock protein transcripts, which are molecular biomarkers for suborganismal protein damage. Frozen larvae tended to have a higher abundance of two transcripts, *hsp60* and *hsp90* (Figure 3c,e). In the case of *hsp60*, transcript levels tended to decrease in supercooled larvae but remained higher in frozen larvae across the various treatments. In the case of *hsp90*, there was a significant effect of treatment in our full factorial model, once again, with frozen larvae tending to have higher expression. Interestingly, these two transcripts were also elevated in frozen larvae relative to those that had enhanced freezing tolerance via rapid cold hardening [24], suggesting that *hsp60* and *hsp90* may be biomarkers of freezing stress. While there was no general treatment effect for *hsp70*, transcript levels were higher in frozen larvae following 12 h recovery from a 24 h cold exposure. This transcript was also differentially expressed between frozen and supercooled larvae in our previous study using Northern blot analyses [21]. The transcript for *sHsp* was the most responsive to cold exposure, but its expression did not differ between frozen and supercooled larvae across our treatments (Figure 1a). Overall, while our effect sizes were relatively small, we did observe higher levels of certain transcripts in frozen larvae, suggesting increased levels of protein damage.

While our results clearly indicate that nonlethal freezing is costly for winter acclimated larvae, we acknowledge that our experiments may not accurately reflect field conditions. The presence of environmental ice in the field may favor cryoprotective dehydration [19], thus it is unclear whether prolonged supercooling is ever used in the field. However, if cooling rates are too fast to permit dehydration, supercooling is likely used, at least for short periods of time. Thus, our 24 h supercooling treatment is likely ecologically relevant. Supercooling for 2 weeks without dehydrating may be unlikely, but we included this longer treatment because we were primarily interested in identifying physiological challenges associated with freezing rather than simulating natural conditions. Furthermore, it is still unclear the extent to which cryoprotective dehydration is used in the field. Gradual chilling from 0 to −3 °C over several days induces cryoprotective dehydration in the laboratory [18,19], but whether cryoprotective dehydration is possible during sudden cold snaps has not been addressed. Further, while cryoprotective dehydration has been observed in artificially rehydrated soils [19], later studies were unable to replicate this result [20]. Thus, the relative probabilities of freezing, supercooling, or undergoing cryoprotective dehydration in the field are uncertain and warrant further investigation. 

## 5. Conclusions

The midge *Belgica antarctica* is the world’s southernmost insect and is faced with the threat of cold stress year-round. While microhabitat temperatures rarely reach the supercooling point [18,19,35], larvae are at high risk of inoculative freezing in water-saturated environments [20]. In earlier work, we showed that at the same temperature (−5 °C), summer-acclimatized larvae fare much better when supercooled than when frozen [21]. Here, with winter-acclimated midges, larvae survived cold exposure up to 14 days equally well, whether frozen or supercooled, and there was no evidence of sublethal tissue damage. However, there was a slight energetic cost to being frozen, and elevated transcript levels of certain heat shock proteins in frozen larvae suggest greater cell stress. We worked with larvae that were stored in our home laboratory, thus in future studies, it would be useful to conduct similar experiments in freshly collected larvae to determine if similar costs of freezing exist in the field. Nonetheless, these results suggest that freezing is costly for winter acclimated larvae, which may favor the selection of dry overwintering environments where it is possible to avoid freezing. However, the Antarctic Peninsula is experiencing increased precipitation in response to climate change [36], which may put larvae at increased risk of inoculative freezing. Furthermore, climate change is increasing thermal variability [37,38], which may further increase the frequency of freezing events. The Antarctic midge and other terrestrial arthropods have persisted in Antarctica since its split from South America ~30 million years ago [39] but increased the risk of inoculative freezing due to climate change may provide a challenge to these ecosystems.

## Figures and Tables

**Table 1:** Primers used for qPCR.

Gene	GenBank Accession	Forward Primer	Reverse Primer
*rpl19*	JX462670	5′-ACATCCACAAGCGTAAGGCTGAGA-3′	5′-TTCTTGTTTCTTGGTGGCGATGCG-3′
*sHsp*	GAAK01009816	5′-GACACCCTTATCAGACGACTAC-3′	5′-CTTCTCGTTCTTCGTGCTTTG-3′
*Hsp40*	GAAK01004380	5′-ACTCTGACCGGAGAAGTGATA-3′	5′-CTCGCTTTGTTGGCTCTTTG-3′
*Hsp60*	GAAK01010161	5′-GTTGCAGGGAGTTGACATAC-3′	5′-GGCAACAGTTACACCATCTT-3′
*Hsp70*	GAAK01011953	5′-CTGCTTTGGCTTACGGTTTG-3′	5′-CCTTCGTCGATGGTCAAGATAG-3′
*Hsp90*	GAAK01011429	5′-CCGGTGGTAGCTTTATCATCTC-3′	5′-GGTAACGATGGCCTTGATCTTA-3′
